# Qianliening capsule treats benign prostatic hyperplasia via suppression of the EGF/STAT3 signaling pathway

**DOI:** 10.3892/etm.2013.1008

**Published:** 2013-03-15

**Authors:** JIUMAO LIN, JIANHENG ZHOU, WEI XU, XIAOYONG ZHONG, ZHENFENG HONG, JUN PENG

**Affiliations:** 1 Academy of Integrative Medicine Biomedical Research Center, Fujian University of Traditional Chinese Medicine, Minhou Shangjie, Fuzhou, Fujian 350122, P.R. China; 2 Fujian Key Laboratory of Integrative Medicine on Geriatrics, Fujian University of Traditional Chinese Medicine, Minhou Shangjie, Fuzhou, Fujian 350122, P.R. China; 3 Departments of Integrative Medicine, Fujian University of Traditional Chinese Medicine, Minhou Shangjie, Fuzhou, Fujian 350122, P.R. China; 4 Pharmacology, Fujian University of Traditional Chinese Medicine, Minhou Shangjie, Fuzhou, Fujian 350122, P.R. China

**Keywords:** qianliening capsule, benign prostatic hyperplasia, epidermal growth factor, signal transducer and activator of transcription 3

## Abstract

Benign prostatic hyperplasia (BPH) is a pathological overgrowth of the human prostate. It may cause increased resistance to urine flow through the urethra and occasionally kidney damage, bladder stones and urinary tract infections, and therefore affect the quality of life. Qianliening capsule (QC) is a traditional Chinese formula that has been used clinically in China to treat BPH for a number of years. However, the mechanism of its anti-BPH effect remains largely unknown. We evaluated the therapeutic effect of QC in a rat model of BPH, established by the injection of testosterone following castration, and investigated the underlying molecular mechanism of action. We observed that QC treatment significantly and dose-dependently decreased the prostatic volume (PV) and prostatic index (PI; P<0.05 or P<0.01), and ameliorated the histological damage of the prostate tissue in the BPH rats. In addition, treatment with QC inhibited the phosphorylation of signal transducer and activator of transcription 3 (STAT3), as well as the expression of epidermal growth factor (EGF), epidermal growth factor receptor (EGFR), cyclin D1 and Bcl-2. Our results suggest that suppression of the EGF/STAT3 pathway may be one of the mechanisms by which QC treats BPH.

## Introduction

Benign prostatic hyperplasia (BPH), a noncancerous enlargement of the prostate gland, is a common prostate disorder among older men. An estimated 50% of men have histological evidence of BPH by age 50 years and 80% by age 70 (1). With the prolonged average life span, increasing elderly population and increasing incidence, BPH has become a major disease of significant interest. The enlarged prostate gland puts pressure on the urethra and/or causes the muscles around the urethra to contract, resulting in partial, or sometimes virtually complete, obstruction of the urethra, which interferes with the normal flow of urine. BPH leads to lower urinary tract symptoms (LUTS) including urinary hesitancy, frequent urination, urgency, thin urine flow and urinary retention (2). These symptoms greatly affect the physical and mental health of patients, as well as their quality of life. Delayed treatment causes numerous severe complications, including bleeding from the prostate, recurrent infections, renal stones and even kidney failure.

Although the pathogenesis of BPH is complex and remains unclear, the important role of androgens, including testosterone and its metabolite dihydrotestosterone (DHT), in the progression of BPH is well established (3). Testosterone or DHT exerts its function by binding to nuclear androgen receptors that are located in the surfaces of stromal cells and epithelial cells, which in turn promotes the transcription of growth factors, including epidermal growth factor (EGF) (4–6). EGF is an important mitosis- and proliferation-promoting factor that has been shown to play a critical role in the development of the prostate after binding to its specific receptor (EGFR, a receptor protein tyrosine kinase) (5–8). Upon interaction with EGF, EGFR induces the phosphorylation and activation of signal transducer and activator of transcription 3 (STAT3), a transcription factor essential for cell survival and proliferation. The phosphorylation of STAT3 in the cytoplasm induces its homodimerization, nuclear translocation and DNA binding, resulting in the expression of genes that mediate proliferation (e.g. cyclin D1) and prevent apoptosis (e.g. Bcl-2) (9). Abnormal activation of the EGF/STAT3 pathway causes an increase in the total number of stromal and epithelial cells, which is strongly associated with the development of BPH (10–15).

At present, pharmacotherapy remains the modality of choice for BPH treatment, and may be roughly divided into three groups: α-blockers, 5α-reductase inhibitors and alternative therapies. The α-blockers, including terazosin, doxazosin and tamsulosin (16,17), inhibit α-adrenergic receptors, thereby relaxing smooth muscle in the prostate and the bladder neck and alleviating the restriction of urine flow. The 5α-reductase inhibitors, including finasteride and dutasteride, suppress 5α-reductase, thereby inhibiting DHT production and the enlargement of prostate. However, these prescription medications may have adverse side effects, including orthostatic hypotension, decreased libido and ejaculatory or erectile dysfunction (18–22). Due to these risks, natural products that appear to have limited adverse events are becoming increasingly important in the treatment of BPH. Although the mechanisms of action are unknown, herbal remedies, including saw palmetto, *Pygeum africanum* and *Hypoxis rooperi*(23–25), have long been used to treat BPH successfully.

Qianliening capsule (QC) is a traditional Chinese medicine formulation that consists of a combination of five natural products (Table I), including rhubarb, leech, Astragalus, Achyranthes and Dodder. These products together confer properties of heat-clearing, detoxification, promotion of blood circulation, removal of blood stasis, tonifying the kidney and nourishing vitality (replenishing the kidney qi in Chinese medicine) (26,27). In the past two decades, QC has been shown to have significant therapeutic effects on BPH (27–29). In clinical trials, QC markedly improved BPH symptoms, by increasing the free maximum urinary flow rate and average urinary flow rate, alleviating frequent urination and urinary urgency, and improving the dynamic index of urine flow (30). In addition, in tests with experimental animals, QC significantly decreased the prostatic volume and weight, inhibited prostatic hyperplasia, attenuated the abnormal serum levels of estrogen and androgen, regulated the expression of estrogen receptor (ER), androgen receptor (AR) and related mRNA, and inhibited the expression of pro-proliferative PCNA, cyclin D1 and CDK4 in the prostatic tissues of BPH rats (26–30). However, the mechanism of its anti-BPH activity remains largely unknown. Therefore we evaluated the effect of QC in a rat model of BPH, established by the injection of testosterone following castration, and investigated the underlying molecular mechanism.

## Materials and methods

### Animals

Sixty SPF grade male Sprague-Dawley (SD) rats (with an initial body weight of 200–220 g) were purchased from Shanghai Si-Lai-Ke Experimental Animal Ltd. (Shanghai, China). The rats were housed in clean pathogen-free rooms in an environment with controlled temperature (22°C), humidity and a 12 h light/dark cycle with free access to water and standard laboratory food. All animal treatment was strictly in accordance with international ethical guidelines and the guide for the Care and Use of Laboratory Animals (31), and the experiments were approved by the Institutional Animal Care and Use Committee of Fujian University of Traditional Chinese Medicine (Fuzhou, China).

### Drugs and reagents

QC (Fujian, China, FDA approval No.: Z09104065) is a capsule of five Chinese products, as listed in Table I, that was provided by the Academy of Pharmacology of Fujian University of Traditional Chinese Medicine. The drug powder inside the capsule was dissolved in distilled water and stored at 4°C. Testosterone propionate injection solution (25 mg/ml) was obtained from Shanghai GM Pharmaceutical Co., Ltd. (Shanghai, China; batch number: H31020524). Finasteride was obtained from Merck (Hangzhou, China; batch number: J20050041). TRIzol reagent was purchased from Invitrogen (Carlsbad, CA, USA). SuperScript II reverse transcriptase was obtained from Promega (Madison, WI, USA). Rat EGF ELISA kit was obtained from Shanghai Xitang Biological Technology Ltd. (Shanghai, China). EGF, EGFR, p-STAT3, Bcl-2 and cyclin D1 primary antibodies, secondary antibody, streptavidin-peroxidase (SP) and 3,3’-diaminobenzidine (DAB) were purchased from Bohai Biotechnology Development Co., Ltd. (Shijiazhuang, China). All other chemicals, unless otherwise stated, were obtained from Sigma-Aldrich (St. Louis, MO, USA).

### Construction of the rat BPH model and drug administration

The rat model of BPH was induced by the subcutaneous injection of testosterone propionate following castration. The scrota of 50 rats from a total 60 male SD rats were removed. One week after surgery, the rats were randomly divided into six groups (n=10), termed the normal group (saline 10 ml/kg), model group (saline 10 ml/kg), finasteride group (0.5 mg/kg) and three QC groups in which rats were orally treated with 2.25, 4.5 or 9 g/kg of QC. The rats in the treated groups received the corresponding drug dose via gastrogavage, together with a subcutaneous injection of testosterone propionate (5 mg/kg), daily for 28 days. The body weight (BW) was measured once per week.

### Sample collection

At the end of the experiments, the animals were weighed, anesthetized with ketamine-diazepam by intraperitoneal injection and the blood was obtained aseptically from the abdominal aorta. The blood-containing tubes were allowed to stand at room temperature for 2 h and sera were obtained by centrifuging at 3000 × g for 20 min in 4°C and stored in −80°C. The intact prostate tissue was dissociated and removed with caution. The prostate weight (PW) and prostatic volume (PV) were measured and the prostatic index (PI) was calculated as: PW/BW×100. One piece of prostate tissue was collected from the same position and fixed with 10% formalin or stored in liquid nitrogen for further analyses.

### Histopathological examination

Small sections of the prostatic specimens were fixed with 10% buffered formalin for 24 h. The samples were then paraffin-embedded, sectioned and stained with hematoxylin and eosin (H&E). Histopathological changes were observed under a light microscope.

### Detection of EGF level in serum by ELISA

The serum level of EGF was measured using an ELISA kit according to the manufacturer’s instructions. The wells were coated with 100 *μ*l capture antibody diluted in coating buffer. The plate was sealed and incubated overnight at 4°C. After three washes, the wells were blocked with 200 *μ*l assay diluents at room temperature for 1 h, followed by another three washes. Diluted EGF standard (100 *μ*l) and test samples were added and incubated for 2 h at room temperature. After repeated washing, the substrate (O-Phenylenediamine, OPD) was added and incubated for 20 min at room temperature and the absorbance was measured at 450 nm using an ELISA reader (Model ELX800; BioTek, Winooski, VT, USA).

### RNA extraction and RT-PCR analysis

Total RNA was isolated from fresh prostate tissues with TRIzol reagent. Oligo (dT)-primed RNA (1 *μ*g) was reverse-transcribed with SuperScript II reverse transcriptase (Promega) according to the manufacturer’s instructions. The obtained cDNA was used to determine the mRNA levels of EGF, EGFR, Bcl-2 and cyclin D1 by PCR with Taq DNA polymerase (Fermentas, Burlington, Canada). β-actin was used as an internal control. The sequences of the primers used for amplification of EGF, EGFR, Bcl-2, cyclin D1 and β-actin transcripts were as follows: EGF, forward: 5′-GCC AAT GCT CAG AAG GCT AC-3′ and reverse: 5′-CGT AAG TCT CGG TGC TGA CA-3′ (temperature=55°C, 361 bp); EGFR, forward: 5′-TCG GTG CTG TGC GAT TTA-3′ and reverse: 5′-TTT CTG GCA GTT CTC CTC-3′ (temperature=50°C, 194 bp); Bcl-2, forward: 5′-GGT GGT GGA GGA ACT CTT CA-3′ and reverse: 5′-GAG CAG CGT CTT CAG AGA CA-3′ (temperature=56°C, 268 bp); cyclin D1, forward: 5′-GGA GCA GAA GTG CGA AGA-3′ and reverse: 5′-GGG TGG GTT GGA AAT GAA-3′ (temperature=57°C, 394 bp); β-actin, forward: 5′-ACT GGC ATT GTG ATG GAC TC-3′ and reverse: 5′-CAG CAC TGT GTT GGC ATA GA-3′ (temperature=55°C, 453 bp). The samples were analyzed by gel electrophoresis (1.5% agarose). The DNA bands were examined using a Gel Documentation system (Bio-Rad, Hercules, CA, USA; Model Gel Doc 2000).

### Immunohistochemical (IHC) analysis

A 0.5×0.5×0.1 cm block of tissue was collected from the lateral lobe of the prostate gland of each rat. Tissue blocks were rinsed with phosphate-buffered saline (PBS), fixed with 10% formaldehyde for 12–24 h, embedded in paraffin, archived and sliced. The paraffin sections were used for EGF, EGFR, p-STAT3, Bcl-2 and cyclin D1 IHC staining. The primary antibodies employed were polyclonal rabbit anti-rat EGF, EGFR, p-STAT3, Bcl-2 and cyclin D1. PBS was used to replace the primary antibody as a negative control. Color was developed using DAB chromogen, as per the manufacturer’s instructions. After staining, five high-power fields (magnification, ×400) were randomly selected in each slide, and the average proportions of positive cells in each field were counted using the true color multi-functional cell image analysis management system (Image-Pro Plus, Media Cybernetics, Rockville, MD, USA).

### Statistical analysis

Data are expressed as mean ± standard deviation (SD). The comparisons between the six groups were performed using the Kruskal-Wallis test and the comparisons between two groups were conducted using the Mann-Whitney U test. For categorical variables, data are presented by number and percentage. The associations between categorical variables were tested using Fisher’s exact test. P<0.05 was considered to indicate a statistically significant result. Statistical analyses were performed using SPSS 15.0 statistics software (SPSS Inc, Chicago, IL, USA).

## Results

### Effects of QC on BW, PW, PV and PI

**Figure 1:**
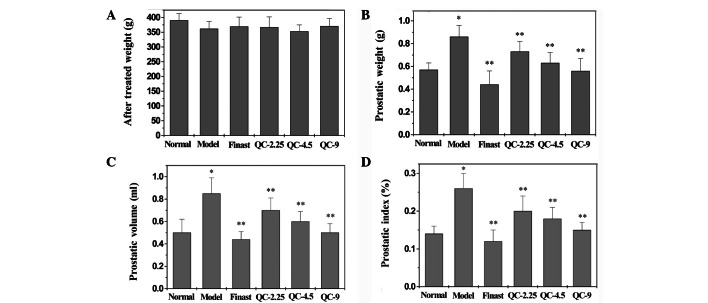
Effect of QC treatment on BW, PW, PV and PI. After treatment (A) body weight (BW); (B) prostatic weight (PW); (C) prostatic volume (PV); and (D) prostatic index (PI). Data are averages with SD (error bars). ^*^P<0.01, vs. normal; ^**^P<0.05, vs. model. QC, Qianliening capsule (g/kg); Finast, finasteride.

We monitored whether QC or finasteride treatment caused any adverse health effects during the study by measuring BW gain, which is a relevant and widely used primary indicator for assessing the gross toxicity of drugs in intervention studies. As shown in Fig. 1A, oral administration of QC and finasteride did not affect BW gain (P>0.05, versus control group), which was consistent with our previous study of toxicity (32). In the model group, the PW, PV and PI increased significantly compared with those in the normal group (P<0.05; Fig. 1B–D), and remained elevated for a continuous period of 28 days, indicating successful model construction. However, treatment with finasteride or different doses of QC significantly reduced the PW, PV or PI in BPH rats compared with those in the model group (P<0.05; Fig. 1B–D). These findings suggest that QC has comparable efficacy to finasteride in the treatment of BPH in rats, without any apparent signs of toxicity.

### QC treatment ameliorates the damage to prostate tissue

**Figure 2:**
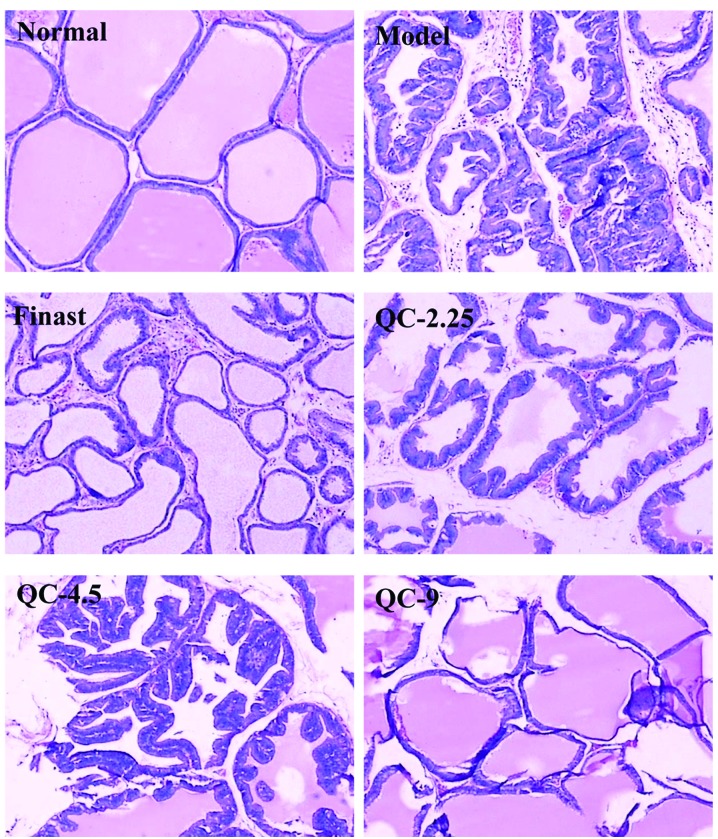
Effect of QC treatment on the histopathological changes in prostate tissue. Samples were stained with hematoxylin and eosin (H&E) and observed under a microscope. Images are representative photographs, magnification ×100. QC, Qianliening capsule (g/kg); Finast, finasteride.

In the normal group, low columnar epithelial cells were arranged as a single layer forming a secretary lumen which was filled with thin acidophilic materials. In the model group, the epithelial cells proliferated markedly to develop excessive glands and cells were arranged as multiple unorganized layers. In all treated groups, the cell proliferation and gland development were significantly inhibited. In addition, QC treatment ameliorated the histopathological changes in a dose-dependent manner (Fig. 2).

### QC downregulates the expression of EGF and EGFR in BPH rats

**Figure 3:**
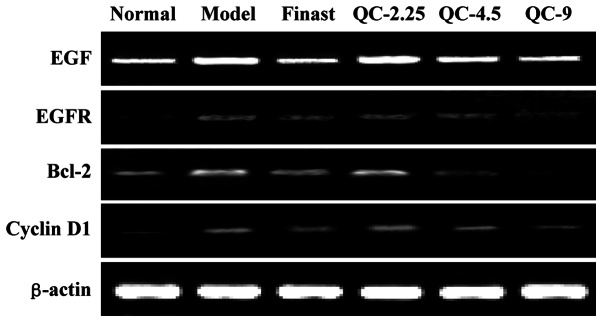
Effect of QC treatment on the mRNA expression of EGF, EGFR, Bcl-2 and cyclin D1 in prostatic tissue. The mRNA levels of EGF, EGFR, Bcl-2 and cyclin D1 in prostatic tissue were determined by RT-PCR and shown by electrophoresis. β-actin was used as an internal control. QC, Qianliening capsule (g/kg); Finast, finasteride; EGF, epidermal growth factor; EGFR, epidermal growth factor receptor.

**Figure 4:**
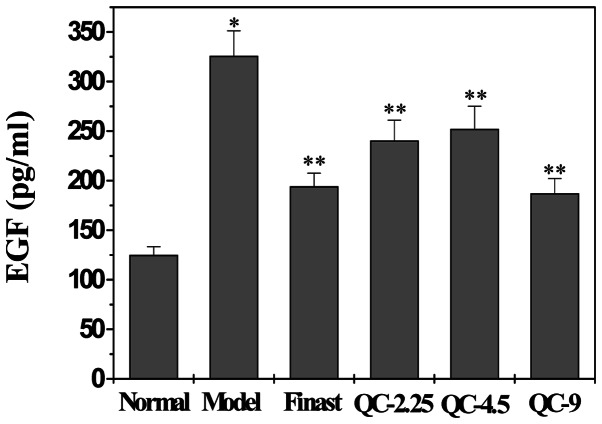
Effect of QC treatment on the secretion level of EGF in serum. The levels of EGF were examined by ELISA. Data are averages with SD (error bars). ^*^P<0.01, vs. normal; ^**^P<0.05, vs. model. QC, Qianliening capsule (g/kg); Finast, finasteride; EGF, epidermal growth factor.

**Figure 5:**
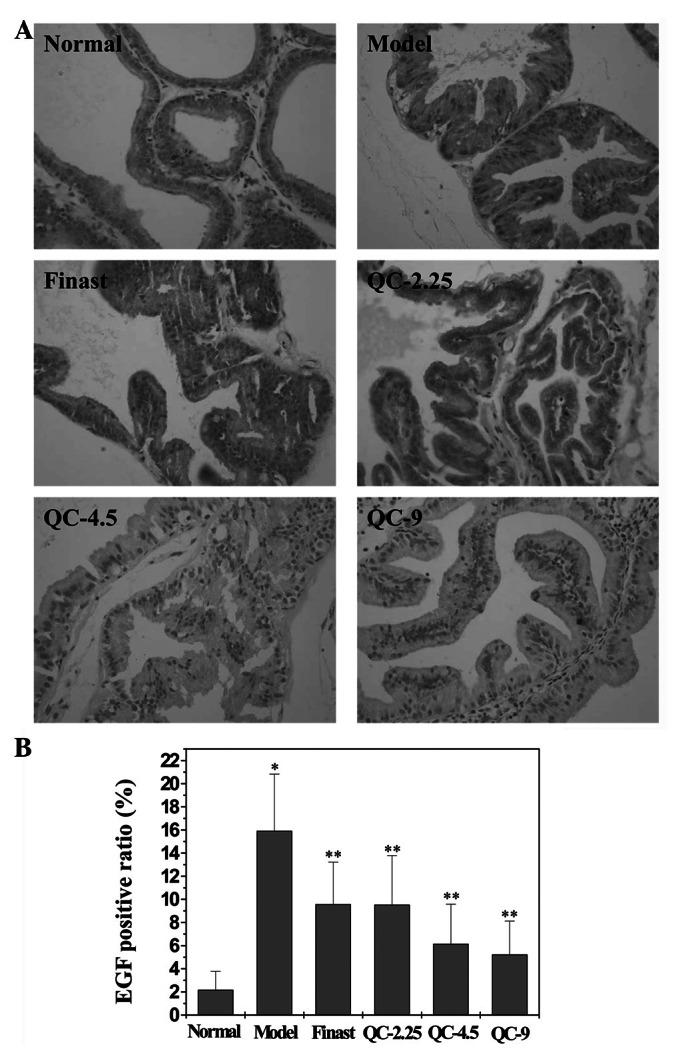
Effect of QC treatment on the protein expression of EGF in prostatic tissue. (A) The protein expression of EGF was observed using IHC staining (magnification ×400). (B) The average proportion of positive cells in each field was counted using the true color multi-functional cell image analysis management system. Data are averages with SD (error bars). ^*^P<0.01, vs. normal; ^**^P<0.01, vs. model. QC, Qianliening capsule (g/kg); Finast, finaste-ride; EGF, epidermal growth factor; IHC, immunohistochemical.

**Figure 6:**
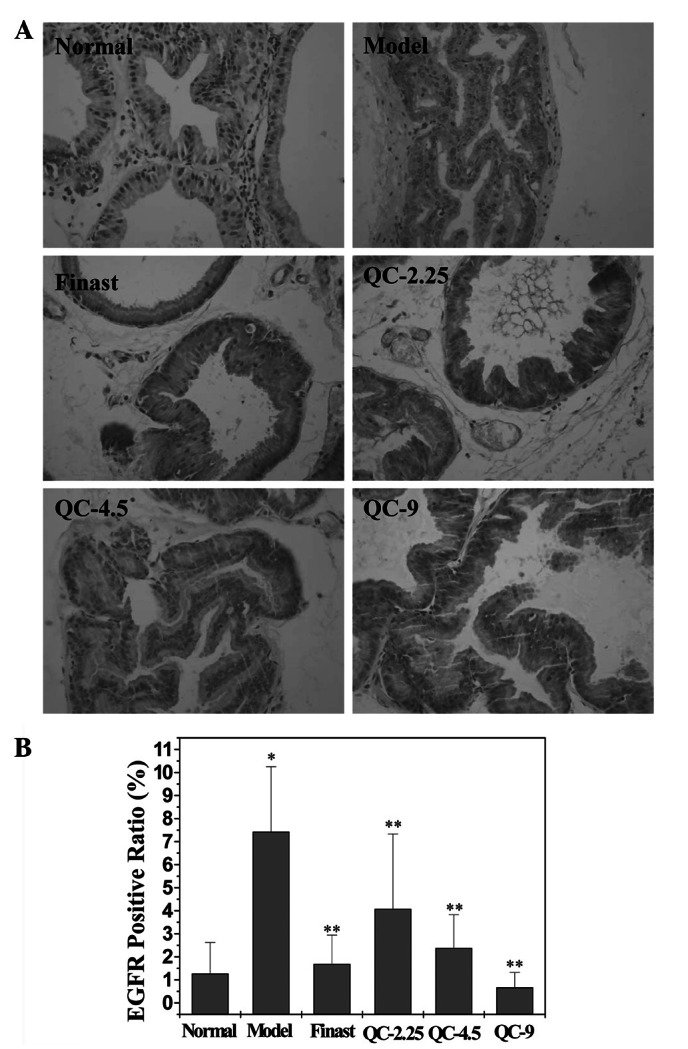
Effect of QC treatment on the protein expression of EGFR in prostatic tissue. (A) The protein expression of EGFR was observed using IHC staining (magnification ×400). (B) The average proportion of positive cells in each field were counted using the true color multi-functional cell image analysis management system. Data are averages with SD (error bars). ^*^P<0.01, vs. normal; ^**^P<0.01, vs. model. QC, Qianliening capsule (g/kg); Finast, finasteride; EGFR, epidermal growth factor receptor; IHC, immunohistochemical.

The mRNA or protein expression of EGF and EGFR in the prostatic tissue of BPH rats was detected using RT-PCR or IHC analysis, respectively, and the secretion level of EGF in serum was examined by ELISA. The results of the RT-PCR assay showed that the mRNA expression levels of EGF and EGFR in the model group were significantly increased, compared with those in the normal group (P<0.05), and these elevations were attenuated by treatment with finasteride or different doses of QC (Fig. 3). Data from IHC analysis and ELISA showed that the pattern of protein expression of EGF and EGFR was similar to that of their respective mRNA levels (Figs. 4–6).

### QC suppresses the STAT3 signaling pathway in BPH rats

**Figure 7:**
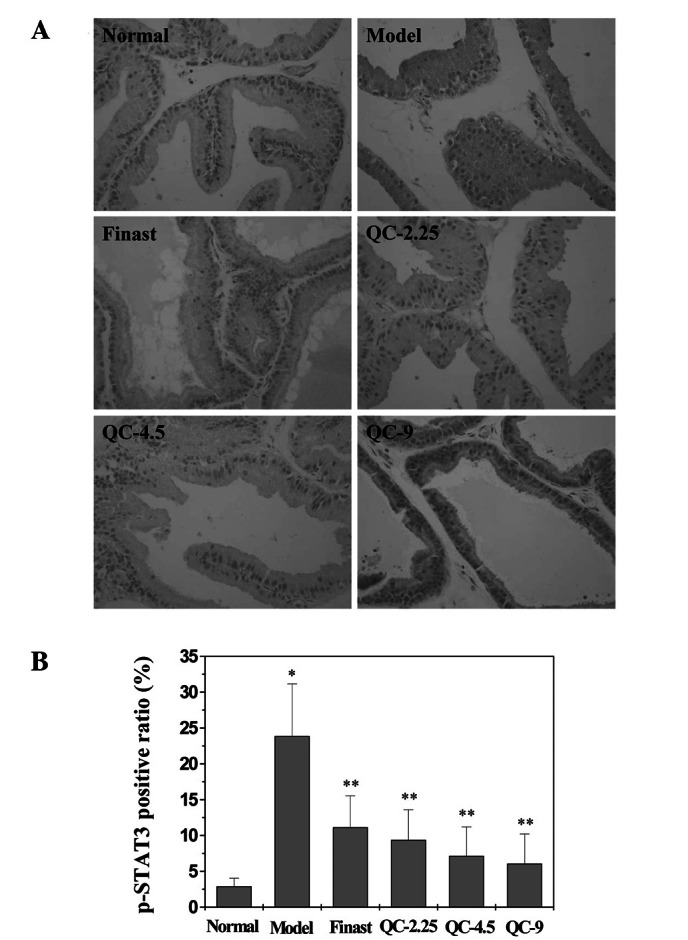
Effect of QC treatment on STAT3 phosphorylation in prostatic tissue. (A) The protein expression of phosphorylated STAT3 (p-STAT3) was detected by IHC staining (magnification ×400). (B) The average proportion of positive cells in each field was counted using the true color multi-functional cell image analysis management system. Data are averages with SD (error bars). ^*^P<0.01, vs. normal; ^**^P<0.01, vs. model. QC, Qianliening capsule (g/kg); Finast, finasteride; IHC, immunohistochemical; STAT3, signal transducer and activator of transcription 3.

**Figure 8:**
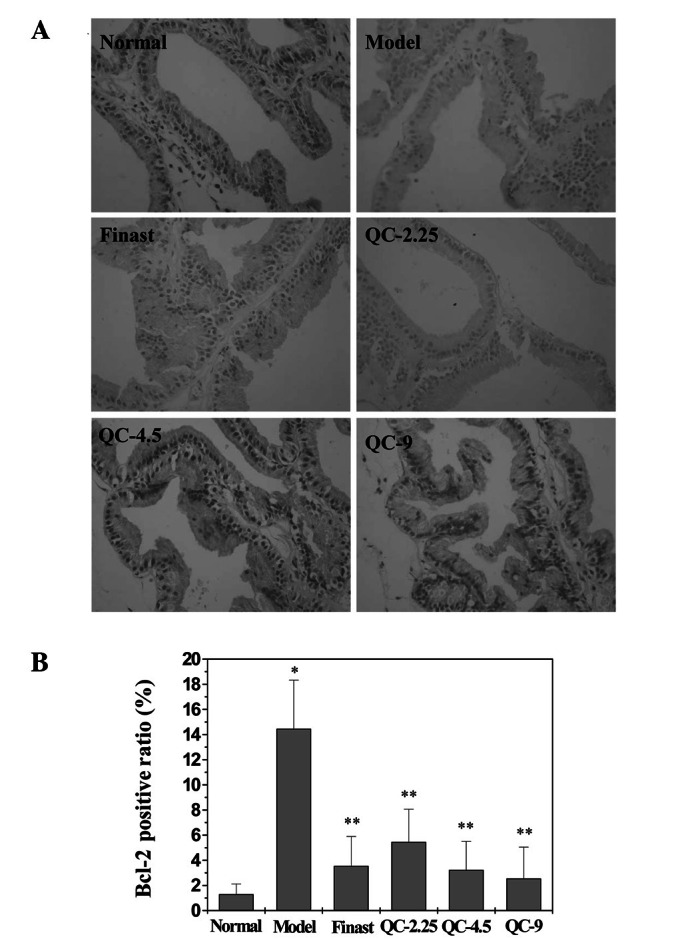
Effect of QC treatment on the protein expression of Bcl-2 in prostatic tissue. (A) The protein expression of Bcl-2 was detected by IHC staining (magnification ×400). (B) The average proportion of positive cells in each field was counted using the true color multi-functional cell image analysis management system. Data are averages with SD (error bars). *P<0.01, vs. normal; ^**^P<0.01, vs. model. QC, Qianliening capsule (g/kg); Finast, finasteride; IHC, immunohistochemical.

**Figure 9:**
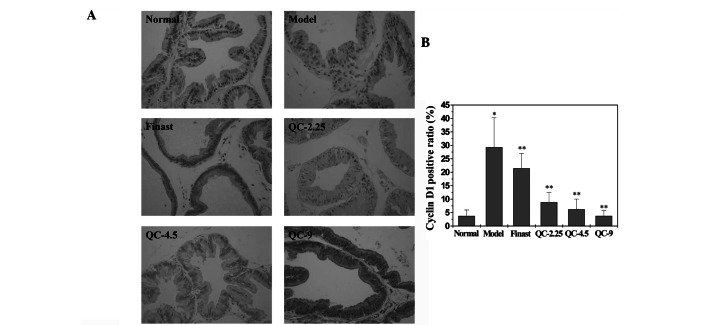
Effect of QC treatment on the protein expression of cyclin D1 in prostatic tissue. (A) The protein expression of cyclin D1 was detected by IHC staining (magnification ×400). (B) The average proportion of positive cells in each field was counted using the true color multi-functional cell image analysis management system. Data are averages with SD (error bars). ^*^P<0.01, vs. normal; ^**^P<0.01, vs. model. QC, Qianliening capsule (g/kg); Finast, finasteride; IHC, immunohistochemical.

STAT3 phosphorylation in the prostatic tissue of BPH rats was determined using an IHC assay. As shown in Fig. 7, the positive expression level of phosphorylated STAT3 (p-STAT3) in the model group was markedly increased compared with that in the normal group (P<0.05), but treatment with finasteride or QC significantly inhibited the effect of BPH model construction on the STAT3 phosphorylation. The expression of cyclin D1 and Bcl-2, two important target genes of STAT3 pathway, was also detected by RT-PCR and IHC analysis. As shown in Figs. 3, 8 and 9, finasteride or QC treatment profoundly inhibited the expression of cyclin D1 and Bcl-2 induced by the construction of the BPH model, at the transcriptional and translational levels.

## Discussion

Although surgical therapy is of superior efficacy for aged patients and those with severe heart, lung and kidney dysfunction, pharmacotherapy remains the modality of choice for BPH treatment. Pharmacotherapy may be divided into three groups, including α-blockers, 5α-reductase inhibitors and alternative therapies. The α-blockers inhibit α-adrenergic receptors, thereby relaxing smooth muscle in the prostate and the bladder neck and alleviating the blockage of urine flow. The 5α-reductase inhibitors suppress 5α-reductase, thereby inhibiting DHT production and the enlargement of the prostate. However, both α-blockers and 5α-reductase inhibitors induce unfavorable side effects, including orthostatic hypotension, decreased libido and ejaculatory or erectile dysfunction (18–22). Therefore, the investigation of natural products for BPH treatment is important, since natural medicines usually display fewer adverse effects and have long been used clinically to treat various diseases, including BPH (23,24).

QC is a traditional Chinese medicine formulation composed of rhubarb, leech, astragalus, achyranthes and Dodder. QC has been used clinically in China for several years, displaying a significant efficacy in BPH treatment (26–30). However, the mechanism of its anti-BPH activity remains largely unknown.

In the present study, we demonstrated that QC significantly reduced the PI and alleviated the prostatic hyperplasy in BPH rats, indicating its anti-BPH efficacy. In addition, the administration of QC did not affect the BW of the rats, suggesting that QC has no apparent toxicity. Although the cause of BPH is not well understood, it is generally considered that excessive cell proliferation and/or reduction of cell apoptosis, usually resulting from the abnormal activation of the EGF/STAT3 pathway, plays a critical role in the development of BPH. EGF exerts its biological function through binding to its specific receptor, EGFR, that is mainly present in epithelial cells on the basal layer of the prostate gland. It has been shown that the expression of EGF and EGFR is increased in the prostatic tissue of patients with BPH (4,8), suggesting that the overexpression of EGF and EGFR participates in the epithelial cell proliferation process in patients with BPH. In addition to directly promoting proliferation through binding to EGFR, EGF inhibits the TGF-β-mediated apoptosis of prostate cells (33). Our data indicated that QC treatment may significantly decrease the serum level of EGF in BPH rats, as well as downregulate the mRNA and protein levels of EGF and EGFR in prostatic tissue.

STAT3 is a member of the STAT family of transcription factors. STAT3 is activated by numerous growth factors and cytokines, including EGF (27). Activation of STAT3 activates a variety of genes, including Bcl-2 and cyclin D1 (9,34–36). Bcl-2 is an apoptosis inhibitory factor which inhibits programmed cell death (7). Bcl-2 is present in normal basal epithelial cells of the prostate gland and is expressed at significantly higher levels in BPH compared with normal prostatic tissue. Cyclin D1 is a key regulatory protein promoting cell cycle progression from the G1 to the S phase. The IHC data in this study showed that QC lowers the expression levels of p-STAT3, Bcl2 and cyclin D1 in the prostatic tissues of BPH rats.

In summary, we report that inhibition of the EGF/STAT3 pathway may be one of the mechanisms by which QC treats BPH.

## Figures and Tables

**Table I:** Composition of Qianliening capsule (QC).

Common name	Latin name	Part used	Daily adult dose (g)
Rhubarb	*Radix et Rhizoma Rhei*	Dried root	15
Leech	*Hirudo*	Dried body	3
Astragalus	*Radix Astragali*	Dried root	12
Achyranthes	*Radix Achyranthis Bidentatae*	Dried root	9
Dodder	*Semen Cuscutae*	Dried seed	6

## Abbreviations:

QC: Qianliening capsule
BPH: benign prostatic hyperplasia
EGF: epidermal growth factor
STAT3: signal transducer and activator of transcription 3
PW: prostatic weight
PV: prostatic volume
PI: prostatic index
SP: streptavidin-peroxidase
